# Age-Related Changes in Nucleus Pulposus Mesenchymal Stem Cells: An In Vitro Study in Rats

**DOI:** 10.1155/2017/6761572

**Published:** 2017-03-15

**Authors:** Yachao Zhao, Zhiwei Jia, Shanshan Huang, Yaohong Wu, Longgang Liu, Linghan Lin, Deli Wang, Qing He, Dike Ruan

**Affiliations:** ^1^Department of Orthopaedics, Navy General Hospital, Beijing, China; ^2^Department of Orthopaedics, The 306th Hospital of People's Liberation Army, Beijing, China; ^3^Department of Laboratory Medicine, Children's Hospital of Hebei Province, Shijiazhuang, Hebei, China

## Abstract

The functions of mesenchymal stem cells (MSCs) appear to decline with age due to cellular senescence, which could reduce the efficacy of MSCs-based therapies. Recently, MSCs have been identified in the nucleus pulposus, which offers great potential for intervertebral disc (IVD) repair. However, this potential might be affected by the senescence of nucleus pulposus MSCs (NPMSCs), but whether or not this exists remains unknown. The aim of this study was to investigate the age-related changes in NPMSCs. NPMSCs isolated from young (3-month-old) and old (14-month-old) Sprague-Dawley rats were cultured in vitro. Differences in morphology, proliferation, colony formation, multilineage differentiation, cell cycle, and expression of *β*-galactosidase (SA-*β*-gal) and senescent markers (p53, p21, and p16) were compared between groups. Both young and old NPMSCs fulfilled the criteria for definition as MSCs. Moreover, young NPMSCs presented better proliferation, colony-forming, and multilineage differentiation capacities than old NPMSCs. Old NPMSCs displayed senescent features, including significantly increased G0/G1 phase arrest, increased SA-*β*-gal expression, decreased S phase entry, and significant p53-p21-pRB pathway activation. Therefore, this is the first study demonstrating that senescent NPMSCs accumulate in IVD with age. The efficacy of NPMSCs is compromised by donor age, which should be taken into consideration prior to clinical application.

## 1. Introduction

Low back pain (LBP) is a leading health concern, which contributes to patients' disability and causes huge socioeconomic burdens [1, 2]. It is estimated that up to 80% of the population experience LBP over their lifetimes, and the prevalence of LBP is rapidly increasing [3, 4]. Although the causes of LBP are complex and unclear, intervertebral disc degeneration (IVDD) is considered a major culprit for this disorder [5]. Current treatment strategies, whether conservative therapies or surgical interventions, merely aim at symptomatic relief rather than targeting the underlying pathogenesis of IVDD [6, 7]. Therefore, as an attractive alternative to traditional options, biological approaches mainly focusing on restoring the structure and function of the intervertebral disc (IVD) are becoming more of interest [7].

Recently, cell therapy based on mesenchymal stem cells (MSCs) has emerged as a promising approach for the treatment of IVDD [6]. Up to now, exogenous MSCs isolated from many tissues, such as bone marrow [8], adipose tissue [9], umbilical cord [10], and synovia [11], have been used for disc repair and regeneration. These MSCs, when transplanted or cocultured with disc cells, could be of benefit for disc repair and regeneration. However, the microenvironment of a degenerative IVD is characterized by nutrient deficiency, hypertonicity, low pH, hypoxia, and high mechanical loading [12], which is not only disadvantageous for resident disc cells, but also a great challenge for the survival and functionality of transplanted MSCs. It has been reported that these factors in the IVD niche can compromise the viability, proliferation, and matrix synthesis of implanted MSCs [13, 14]. Therefore, it is necessary to find other candidates that better fit the harsh microenvironment.

MSCs reside in almost all adult tissues and play a crucial role in maintaining normal tissue homeostasis [15]. There is also evidence supporting the existence of MSCs within different parts of the IVD, such as the nucleus pulposus (NP) [16], the annulus fibrosus (AF) [17], and the cartilage endplate (CEP) [18]. Recently, endogenous MSCs derived from NP tissues have exhibited great potential for disc repair and regeneration. When maintained in the disc mimicking microenvironment, NP MSCs (NPMSCs) have been found to tolerate IVD-like hypertonicity and show greater bioactivity than adipose-derived MSCs in hypoxic and acidic conditions [19–21]. Therefore, stimulation or transplantation of NPMSCs might have advantages over other cell-based therapies for IVDD within the harsh microenvironment of the IVD niche.

Previous studies show that there are significant differences in morphology of bone marrow MSCs (BMSCs) between different age groups [22–24]. The proliferation and multilineage differentiation capacities of BMSCs decline with age [22, 25–27]. Similar findings have also been made in MSCs derived from adipose tissue [28], skeletal muscle [29], articular cartilage [30], and tendon [31], which may be triggered by stem cell aging [32]. Moreover, MSCs are usually exposed to reactive oxygen species, biological toxins, chemical agents, and physical stressors, which can also lead to premature senescence irrespective of donor age [33].

We hypothesize that NPMSCs may experience cellular senescence with age and/or undergo premature senescence in response to various stresses under the harsh disc microenvironment, which may compromise the efficacy of NPMSCs when applied to the treatment of IVDD. In the present study, we aimed to investigate whether NPMSCs senescence exists and to study the age-related changes in the morphology and functions of NPMSCs.

## 2. Results

### 2.1. Characteristics of NPMSCs Growth and Morphology

**Figure 1:**
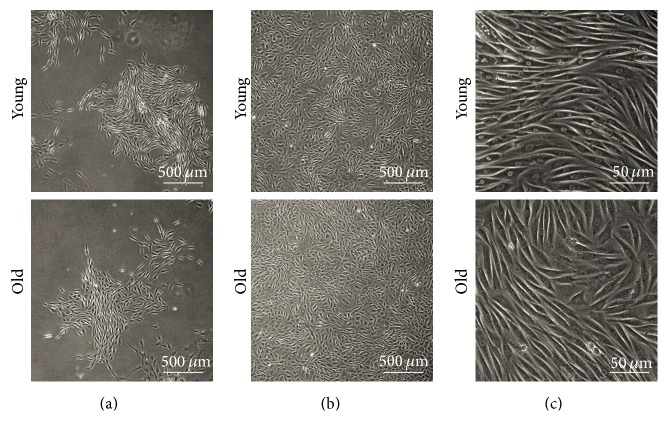
Characterization of NPMSCs growth and morphology (*n* = 5). (a) Larger adherent colonies were observed in young NPMSCs compared to old NPMSCs during primary culture. (b) Young NPMSCs grew more rapidly and reached cell confluence earlier than old NPMSCs after primary isolation. (c) Both young and old NPMSCs at P3 had a small and spindle-shape morphology, with no substantial differences between them.

The yield of NPMSCs isolated from the coccygeal IVD tissues of all 5 old rats was lower than that from young rats. Compared to old NPMSCs, a higher number of plastic adherent colonies were observed in young NPMSCs 3 days after initial seeding (Figure 1(a)). Young NPMSCs grew rapidly and reached over 80% confluence before day 9 after primary isolation (Figure 1(b)). In comparison, adherent colonies only became apparent in old NPMSCs after 6 days following primary isolation (Figure 1(a)). Old NPMSCs expanded slowly and achieved cell confluence by 14 days after initial plating (Figure 1(b)). With regard to cellular morphology, both young and old NPMSCs at P3 exhibited the characteristic small, spindle-shape morphology in culture (Figure 1(c)).

### 2.2. Features of Immunophenotypes

**Figure 2:**
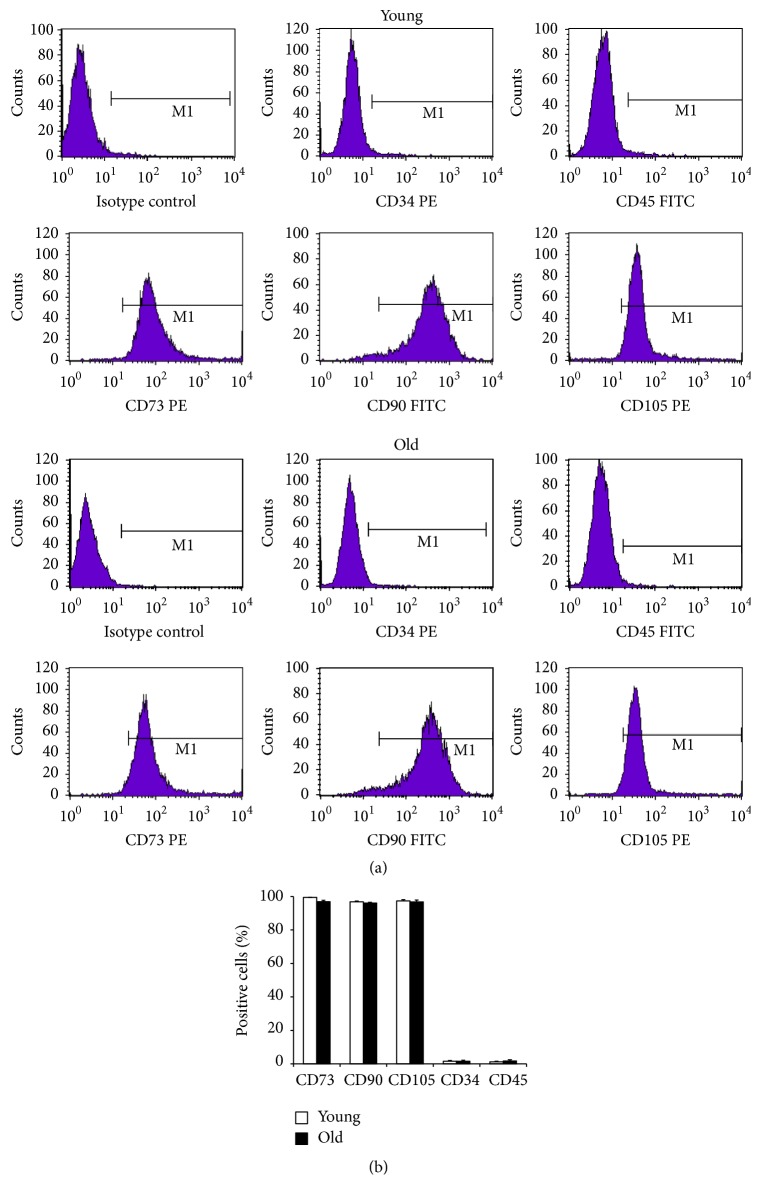
Immunophenotypes of NPMSCs (*n* = 5). (a) Representative graphs of immunophenotypes of young and old NPMSCs detected by flow cytometry. (b) Both young and old NPMSCs were highly (>95%) positive for the MSCs surface markers CD73, CD90, and CD105 and slightly (<5%) positive for the hematopoietic stem cell surface markers CD34 and CD45. The data are expressed as means ± SD.

A series of cell surface antigens commonly used to identify MSCs were analyzed by FACSCalibur (Figure 2(a)). As shown in Figure 2(b), both young and old NPMSCs at P3 were highly positive for the MSCs markers CD73 (*P* = 0.0083), CD90 (*P* = 0.0807), and CD105 (*P* = 0.4918) and negative for the hematopoietic stem cell markers CD34 (*P* = 0.2494) and CD45 (*P* = 0.5127).

### 2.3. Age-Related Decline in Proliferation Capacity

**Figure 3:**
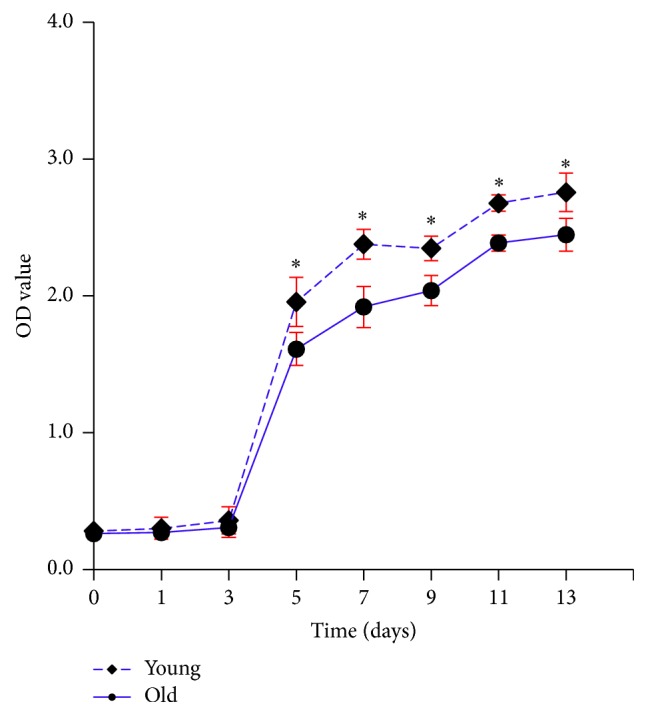
The growth curves of young and old NPMSCs detected by CCK-8 assay (*n* = 5). The OD values of young NPMSCs were significantly stronger than those of old NPMSCs during the logarithmic phase, indicating a better proliferation capacity of young NPMSCs. The data are expressed as means ± SD. *∗* indicates *P* < 0.05.

The CCK-8 assay (Figure 3) showed that both young and old NPMSCs expanded slowly during the initial 3 days after plating, undergoing a slow growth period. After that, NPMSCs proliferated rapidly from day 3 to day 7, entering into a logarithmic phase. Finally, the proliferation rates slowed down again, appearing as an S-shaped growth curve. Furthermore, the optical density (OD) values of young NPMSCs were significantly stronger than of old NPMSCs during the logarithmic phase (*P* = 0.0071 and *P* = 0.0005, day 5 and day 7), indicating a better proliferation capacity in young NPMSCs.

### 2.4. Age-Related Decline in Colony-Forming Ability

**Figure 4:**
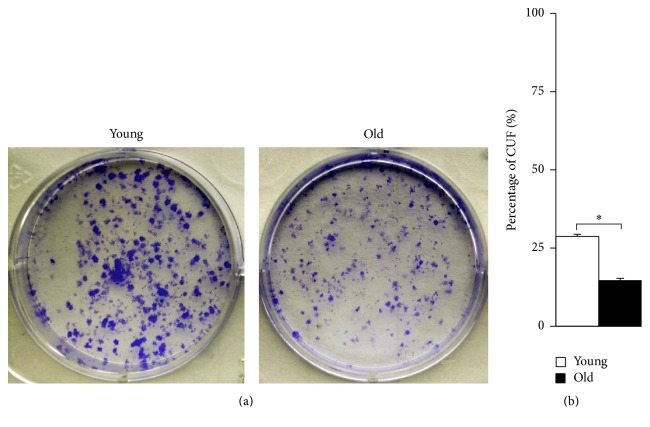
Colony-forming of NPMSCs (*n* = 5). (a) Representative images of Crystal Violet staining of colonies formed in young and old NPMSCs after incubation for 14 days. The colony-forming units (CFUs) formed in young NPMSCs were larger and more numerous than in old NPMSCs. (b) Quantitative analysis also showed that old NPMSCs displayed a significantly lower colony-forming efficiency than young NPMSCs, indicating a better colony-forming ability in young NPMSCs. The data are expressed as means ± SD. *∗* indicates *P* < 0.05.

The colony-forming assay (Figure 4(a)) showed that colonies declined both in size and in number in old compared to young NPMSCs. Further quantitative analysis (Figure 4(b)) also revealed that old NPMSCs exhibited a significantly lower colony-forming efficiency than young NPMSCs (*P* = 0.0000), indicating a better colony-forming ability in young NPMSCs.

### 2.5. Age-Related Decline in Multilineage Differentiation Potential

#### 2.5.1. Adipogenic Differentiation

**Figure 5:**
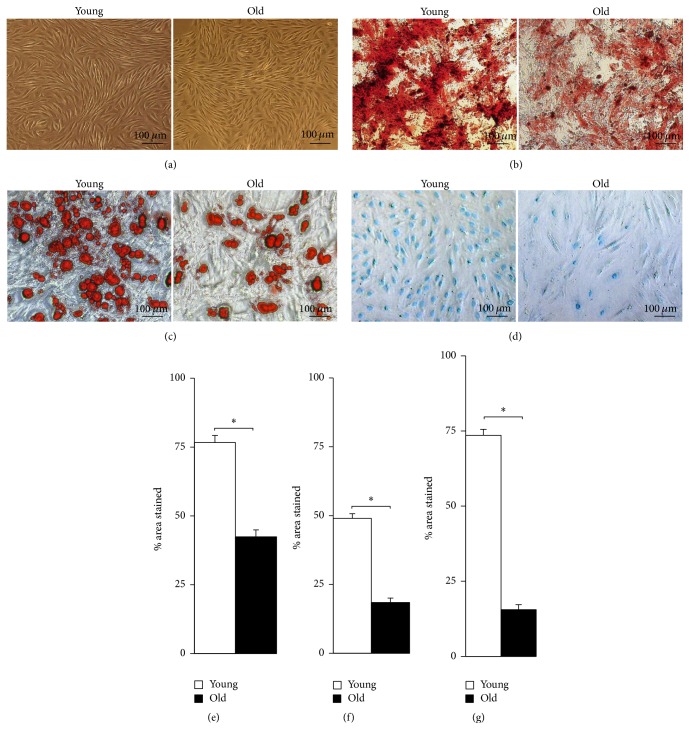
Multipotent differentiation capacities of NPMSCs (*n* = 5). (a) Young and old NPMSCs at P3 served as controls. (b) Osteogenic differentiation of young and old NPMSCs stained with Alizarin Red after 21 days of induction. (c) Adipogenic differentiation of young and NPMSCs stained with Oil Red O after 28 days of induction. (d) Chondrogenic differentiation of young and old NPMSCs stained with Alcian Blue after 21 days of induction. (e) Percentage of total area that was positively stained with Alizarin Red. (f) Percentage of total area that was positively stained with Oil Red O. (g) Percentage of total area that was stained with Alcian Blue. The data are expressed as means ± SD. *∗* indicates *P* < 0.05. A complete decrease in osteogenic, adipogenic, and chondrogenic differentiation capacities was observed in NPMSCs due to increasing donor age.

To assess the adipogenic differentiation potential of young and old NPMSCs, Oil Red O staining was performed to visualize the lipid vacuoles after 4 weeks of induction. Compared to young NPMSCs, a smaller number of old NPMSCs were positively stained with Oil Red O (Figure 5(c)). Quantification was performed by determining the percentage of area that contained Oil Red O-stained lipid vacuoles using ImageJ. As shown in Figure 5(f), this percentage in old NPMSCs was significantly lower than that in young NPMSCs (*P* = 0.0000).

#### 2.5.2. Osteogenic Differentiation

To assess the osteogenic differentiation potential, Alizarin Red staining was performed to visualize calcium deposits after 3 weeks of induction. As shown in Figure 5(b), a large number of old NPMSCs lost their osteogenic differentiation potential, indicating a decrease in osteogenic differentiation potential with increasing age. This was in accordance with the quantitative analysis (Figure 5(e)), which showed that the percentage of area that was stained positively in old NPMSCs was significantly lower compared to young NPMSCs (*P* = 0.0000).

#### 2.5.3. Chondrogenic Differentiation

To assess the chondrogenic differentiation potential, Alcian Blue staining was performed to detect the synthesis of proteoglycans by chondrocytes after 3 weeks of induction. As shown in Figure 5(d), a smaller number of old NPMSCs than young NPMSCs were positively stained with Alcian Blue (Figure 5(g)). Further quantitative analysis also revealed that the percentage of area stained positively was significantly lower in old NPMSCs than that in young NPMSCs (*P* = 0.0000).

### 2.6. Cell Cycle Analysis

**Figure 6:**
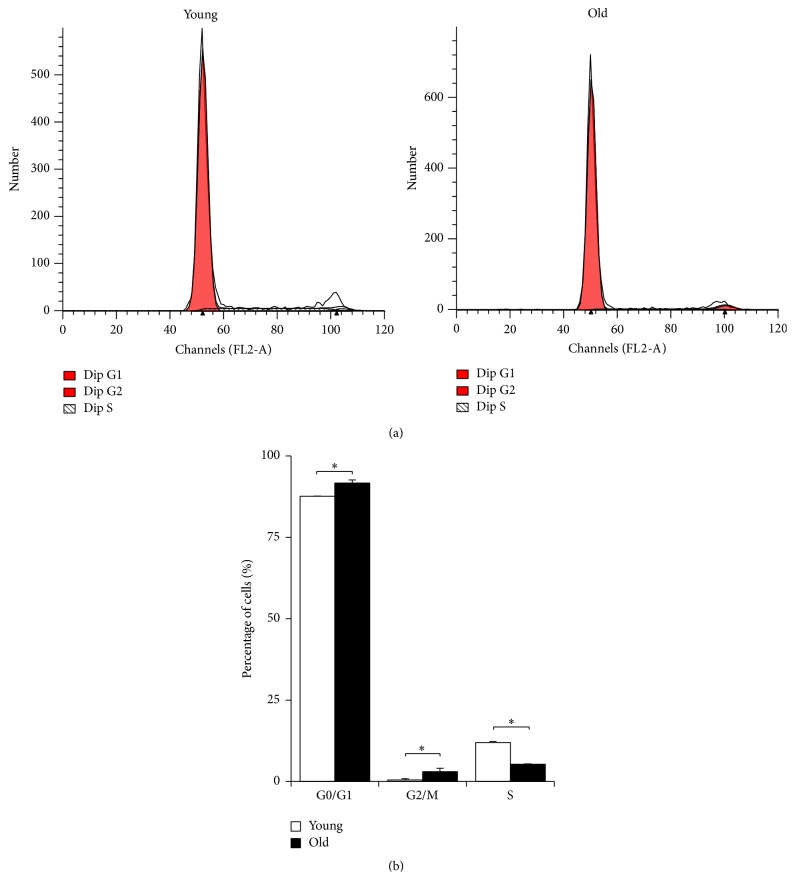
Cell cycles of NPMSCs (*n* = 5). (a) Representative graphs of cell cycles of young and old NPMSCs determined by flow cytometry. (b) Both young and old NPMSCs were mainly in the G0/G1 phase (>80%), but only small percentages of cells were in the G2/M and S phases. Furthermore, increased G0/G1 and G2/M phase arrest coupled with decreased S phase entry was observed in old NPMSCs compared to young NPMSCs. The data are expressed as means ± SD. *∗* indicates *P* < 0.05.

The cell cycle distribution of NPMSCs was also determined using flow cytometry (Figure 6(a)). As shown in Figure 6(b), both young and old NPMSCs were mainly in the G0/G1 phase, whereas only small percentages of each cell population were in the G2/M and S phases. Moreover, old NPMSCs had significantly higher percentages of cells in the G0/G1 and G2/M phases than young NPMSCs (*P* = 0.0495 and *P* = 0.0142, resp.). In contrast, the percentage of cells in the S phase was much lower in old NPMSCs compared to young NPMSCs (*P* = 0.0000).

### 2.7. Age-Related Increase in Cellular Senescence

**Figure 7:**
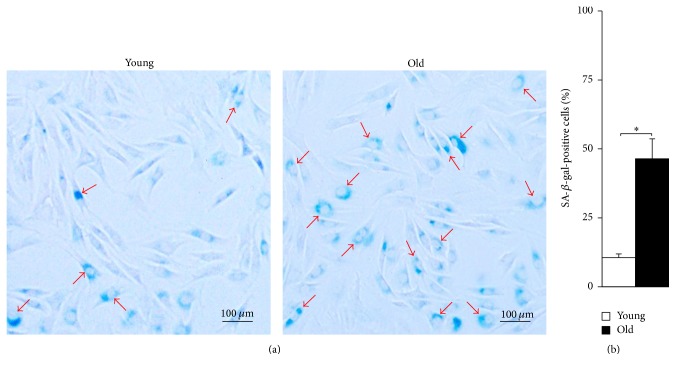
Cellular senescence in NPMSCs (*n* = 5). (a) Representative images of SA-*β*-gal staining for the detection of senescent NPMSCs from young and old rats. An increased number of old NPMSCs were positive (blue color) for SA-*β*-gal compared to young NPMSCs. (b) Quantitative analysis also showed that the percentage of SA-*β*-gal-positive old NPMSCs was significantly higher than that of young NPMSCs. The data are expressed as means ± SD. *∗* indicates *P* < 0.05.

The SA-*β*-gal staining (Figure 7(a)) showed that an increased number of old NPMSCs were positive for SA-*β*-gal compared to young NPMSCs. Further quantitative analysis (Figure 7(b)) also revealed that the percentage of SA-*β*-gal-positive old NPMSCs was significantly higher than that of young NPMSCs (*P* = 0.0000).

### 2.8. Quantitative Real-Time Polymerase Chain Reaction (qPCR) Analysis

**Figure 8:**
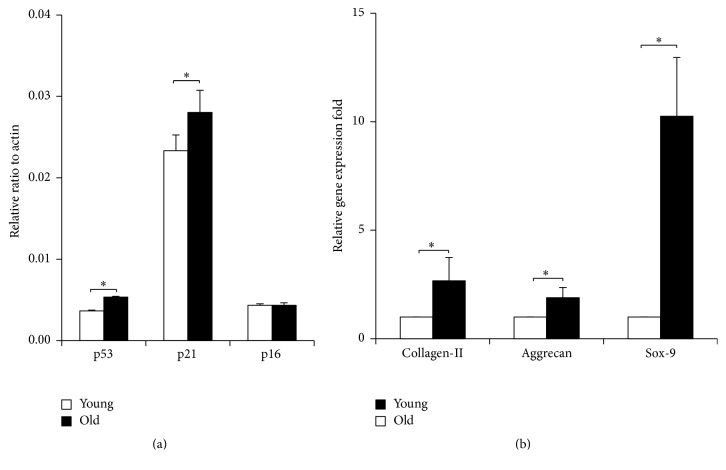
The mRNA expression levels of target genes in NPMSCs analyzed by real-time PCR (*n* = 5). (a) Increased expressions of p53, p21, and p16 were observed in old NPMSCs relative to young NPMSCs. In particular, old NPMSCs expressed significantly higher p53 and p21 than young NPMSCs. (b) Compared with old NPMSCs, young NPMSCs had significantly higher expression levels of collagen-II, aggrecan, and Sox-9. The level of mRNA expression was normalized and graphed relative to *β*-actin. The data are expressed as means ± SD. *∗* indicates *P* < 0.05.

The qPCR analysis (Figure 8(a)) showed an increased expression of p53, p21, and p16 in old NPMSCs compared to young NPMSCs. In particular, old NPMSCs expressed significantly higher p53 and p21 than young NPMSCs (*P* = 0.0000 and *P* = 0.0306, resp.). As shown in Figure 8(b), young NPMSCs had significantly higher expression levels of collagen-II, aggrecan, and Sox-9 than old NPMSCs (*P* = 0.0001, *P* = 0.0001, and *P* = 0.0000, resp.).

## 3. Discussion

MSCs represent a promising cell source for regenerative medicine due to their intrinsic capacities for self-renewal and differentiation into multilineages [34]. MSCs can be isolated from multiple tissues, such as bone marrow [8], adipose tissue [9], umbilical cord blood [10], and synovia [11]. In recent years, NPMSCs have been identified and characterized in NP tissues [35] and have shown great potential for the treatment of IVDD [19–21]. In clinical practice, MSCs are usually applied in the aging population. However, accumulating studies prove that the functions of MSCs, including self-renewal and differentiation, decline with increasing age due to cell senescence [25, 27]. Therefore, the effect of donor age on the functions of NPMSCs has important implications for cell therapy in older patients, who are more likely to be afflicted by IVDD than younger patients. The major findings of our study demonstrated that (i) both young and old NPMSCs fulfilled the requirements for MSCs definition; (ii) young NPMSCs presented a better proliferation capacity and colony-forming ability than old NPMSCs; (iii) the 3-lineage differentiation potential of NPMSCs declined with age; (iv) increased G0/G1 and G2/M phase arrest and decreased S phase entry were observed in old NPMSCs compared with young NPMSCs; and (v) NPMSCs displayed characteristic senescent features with age, including an increase in SA-*β*-gal expression and significant activation of the p53-p21-pRB pathway, which were both associated with cell senescence.

During the initial phases of growth, we observed that both young and old NPMSCs grew by adherence to the plastic surfaces in vitro. With respect to the immunophenotypic pattern, both young and old NPMSCs at P3 highly (>95%) expressed the MSCs markers (CD73, CD90, and CD105) and failed (<5%) to express the hematopoietic markers (CD34 and CD45). Regarding differentiation potential, both young and old NPMSCs could differentiate into multiple lineages, such as osteoblasts, adipocytes, and chondrocytes. Thus, both young and old NPMSCs fulfilled the criteria for definition as MSCs outlined by the International Society of Cell Therapy (ISCT) [36].

With respect to cellular morphology, the cultured NPMSCs from both young and old rats were mainly small and spindle-shaped, which was consistent with the results of Risbud et al. [16]. According to a similar previous report [24], MSCs from aged donors exhibit senescence-like appearances, presenting a spread out, flat, and enlarged morphology with nuclei larger in size than those from young donors. However, we did not observe senescence-like appearances in NPMSCs from old rats. In human NPMSCs from subjects of different ages, Blanco et al. [35] also found no signs of senescence during NPMSCs expansion in vitro over not more than 3 passages. These results may be attributed to the donors not being old enough. In addition, it should be noted that culture passage is another important factor affecting cell aging [26, 27]. If the number of NPMSCs passages was expanded, it may be possible to test this issue.

Previous studies showed that MSCs from aged donors displayed a decline in growth rate and a reduced colony-forming capacity both in size and in number due to cellular senescence [22, 37]. In our study, young NPMSCs expanded more rapidly than old NPMSCs in vitro. In addition, the CCK-8 assay showed that the OD values of young NPMSCs were significantly higher than those of old NPMSCs during the logarithmic growth phase. With respect to the colony-forming analysis, young NPMSCs exhibited a significantly higher colony-forming efficiency than old NPMSCs. These results indicate a better proliferation capacity and colony-forming ability of young NPMSCs. Therefore, our study suggests that the age-related decrease in the self-renewal capacity of NPMSCs may lead to stem cell exhaustion in NP tissue and decrease the self-repair and regenerative potential of IVD.

The differentiation potential of MSCs appears to change with age [38], but there have been controversial results for the effect of donor age on the multipotency of MSCs. Several studies showed that MSCs from aged donors tend to have a reduced capacity for multilineage differentiation [39–41], or even complete abolishment of lineage-specific commitment [24]. In contrast, others found no significant differences in differentiation ability during MSCs aging [42, 43]. In the present study, we also detected a complete decrease in the differentiation potential of osteocytes, chondrocytes, and adipocytes in old NPMSCs. This inconsistency may be attributed to the choice of age groups, group size, isolating and culturing conditions, stimulation of the cells, and tissue-specific divergence. Considering that the chondrogenesis of the transplanted MSCs plays an important role in NP cell activity and proliferation, the decline in chondrogenesis of NPMSCs with advancing age may reduce the potential of NPMSCs to differentiate into NP cells. This may lead to a decrease in the frequency and function of NP cells and consequently decrease biosynthesis of extracellular matrix components. Therefore, these may reduce the efficacy of NPMSCs transplantation for the treatment of IVD degeneration.

SA-*β*-gal is a hallmark to identify senescent cells which can be detected by SA-*β*-gal staining [44]. The present study showed that old NPMSCs possessed significantly higher percentage of SA-*β*-gal-positive cells than young NPMSCs, indicating that senescent NPMSCs may increase or accumulate with advancing age. Considering that stem cells play a crucial role in maintaining normal tissue homeostasis [45], the increase or accumulation of senescent NPMSCs with age may decrease the self-repair and regenerative potential of NP tissues and consequently lead to IVDD. Moreover, the efficacy of transplantation of NPMSCs isolated from aged donors is compromised due to cellular senescence.

We found that the expression of senescence markers (p53, p21, and p16), which are related to replicative [46] and stress-induced senescence pathways [47], increased in rat NPMSCs with advancing age. Compared with young NPMSCs, old NPMSCs expressed significantly higher amounts of p53 and p21, but no difference was observed for p16. These results indicate that both the replicative p53-p21-pRB pathway and the stress-induced p16-pRB pathway are activated simultaneously, but the replicative p53-p21-pRB pathway may play a more important role than the stress-induced p16-pRB pathway in the senescence of rat NPMSCs with advancing age, further confirming that NPMSCs may undergo aging by activation of senescence pathways.

Stem cells are slow-cycling cells characterized by the majority of cells staying in the resting stage (G0/G1 phase), which is closely related to cell pluripotency [48]. In this study, flow cytometry analysis showed similar results. Both young and old NPMSCs were mainly in the G0/G1 phase (>80%), and only small portions of cells were in the proliferative state (G2/M and S phases). Moreover, significantly increased G0/G1 and G2/M phase arrest and decreased S phase entry were observed in old NPMSCs compared to the young group. This result supports the hypothesis that senescent cells remain viable and metabolically active and are permanently growth arrested in either the G0/G1 or the G2/M stage of the cell cycle, which is in accordance with a previous study [49].

There are two major limitations in the present study. First, we selected the 14-month-old rat as old model based on previous reports on other MSCs [24, 26, 39]. However, it was unclear whether the 14-month-old rat was the optimal old model. More data sets especially from older rats should be included in our following work to elucidate this problem. Second, NPMSCs were harvested from SD rats. Future studies on human NPMSCs senescence are needed to provide more clinical relevance.

In summary, this is the first study proving that increased senescence of NPMSCs is present in IVD with advancing age. Considering that stem cells play a key role in maintaining the homeostasis of normal tissue, aging of stem cells in IVD may provide new insight into the mechanisms of IVDD. The understanding of the mechanisms underlying these degenerative processes may be important in helping to improve the prevention and treatment of IVDD. Furthermore, the age-related decline in the functional properties of NPMSCs should be taken into consideration when evaluating the potential utility of autologous NPMSCs isolated from aged donors for regenerative medical therapies.

## 4. Materials and Methods 

### 4.1. Ethics Statements

All experimental protocols below were reviewed and approved by the Laboratory Animal Ethics Committee of Navy General Hospital, Beijing, China, and carried out in accordance with the relevant guidelines and regulations.

### 4.2. Isolation and Culture of NPMSCs

Ten male Sprague-Dawley (SD) rats were included in this study and divided into 2 groups by age: young (3-month-old, *n* = 5) and old (14-month-old, *n* = 5). After being humanely killed by an intraperitoneal overdose injection of 10% chloral hydrate, the coccygeal IVD tissues of all 5 SD rats per group were harvested under aseptic conditions. We further removed the attached muscular and ligamentous tissues and separated the central gelatinous NP tissues from the inner AF by ophthalmic surgical instruments under a sterile dissecting microscope. The isolated NP tissues were washed with phosphate-buffered saline (PBS), minced with the aseptic ophthalmic scissors, and then digested with 0.2% collagenase type II (Sigma-Aldrich, St. Louis, MO, USA) in Dulbecco's modified Eagle's medium-low glucose (DMEM-LG, Solarbio Science & Technology Co., Ltd., Beijing, China) at 37°C for 4 hours. After washing with PBS and centrifuging for 5 minutes at 1500 ×g, the cell pellets were then resuspended in DMEM-LG supplemented with 10% fetal bovine serum (FBS, Gibco BRL, Grand Island, NY, USA) and 1% penicillin-streptomycin. Finally, cells were plated at a density of 2 × 10^5^ cells/mL into 25 cm^2^ cell culture flasks and incubated at 37°C in a 5% CO_2_ humidified incubator (Thermo Fisher Scientific Inc., Waltham, MA, USA). The medium was replaced every 3 days. When adherent cells reached 80%–90% confluence, they were harvested with 0.25% Trypsin-EDTA and subcultured at 1 : 3. Cells at passage 3 (P3) were used for subsequent experiments.

### 4.3. Characteristics of NPMSCs Growth and Morphology

During the primary culture of young and old NPMSCs, we investigated the characteristics of cell growth in vitro, including cell attachment and expansion. In addition, the morphology of NPMSCs at P3 was observed and assessed between the 2 age groups under a phase contrast microscope (Olympus Optical Co. Ltd., Tokyo, Japan).

### 4.4. Identification of Immunophenotypes

Both young and old NPMSCs at P3 were trypsinized, collected, and then washed twice with PBS. After that, 2 × 10^5^ cells were transferred into a 15 mL centrifuge tube and resuspended in 100 *μ*L cold PBS. Next, cells were incubated in the dark for 30 minutes at 4°C with fluorescein isothiocyanate (FITC) or phycoerythrin- (PE-) conjugated antibodies against CD34, CD105 (Abcam, Cambridge, MA, USA), CD45, CD73, and CD90 (BD Pharmingen, San Diego, CA, USA). After that, cells were washed twice with cold PBS, resuspended in 500 *μ*L cold PBS, and then analyzed on a FACSCalibur (BD, USA) for the expression of specific surface markers.

### 4.5. Proliferation Assay

The proliferation capacity of young and old NPMSCs was determined using the Cell Counting Kit-8 (CCK-8, Dojindo Laboratories, Kumamoto, Japan) according to the manufacturer's protocol. Briefly, P3 cells from the 2 ages were plated onto 96-well plates at a density of 1,000 cells/well and then cultured in standard medium. At 1, 3, 5, 7, 9, 11, and 13 days after initial plating, the medium of each well was then gently aspirated and replaced with 110 *μ*L standard medium containing 10 *μ*L CCK-8 solution. After incubation in the dark for 4 hours at 37°C, the OD values were measured at 450 nm using a microplate reader (Model 680, Bio-Rad Laboratories K.K., Tokyo, Japan).

### 4.6. Colony-Forming Assay

For the colony-forming analysis of young and old NPMSCs, P3 cells from the 2 ages were harvested and plated onto a 6-well plate at a density of 1,000 cells/10 cm^2^. The medium was replaced every 3 days. After incubation for 14 days, cells were washed twice with PBS, fixed in 4% paraformaldehyde for 15 minutes, and then stained with 0.1% Crystal Violet (KeyGen Biotech, Nanjing, China) for 15 minutes. Colonies containing more than 30 cells were considered for counting. The efficiency of colony formation was calculated by dividing the number of colonies by the initial number of adherent cells.

### 4.7. Multilineage Differentiation

#### 4.7.1. Adipogenic Differentiation

Young and old NPMSCs were harvested at P3, seeded into a 6-well plate at a density of 2 × 10^4^ cells/cm^2^, and then cultured at 37°C in a 5% CO_2_ humidified incubator. The growth medium was changed every 3 days until cells reached 90% confluence. The medium of differentiated wells was replaced with SD Rat Mesenchymal Stem Cell Adipogenic Differentiation Medium (Cyagen Biosciences, Guangzhou, China) after 4 days following confluence. Briefly, cells were refed with the induction medium A (10% FBS, 1% penicillin-streptomycin, 1% glutamine, 0.2% insulin, 0.1% IBMX, 0.1% rosiglitazone, and 0.1% dexamethasone). After 72 hours of induction, the induction medium was gently aspirated and cells were then cultured for 24 hours in maintenance medium B (10% FBS, 1% penicillin-streptomycin, 1% glutamine, and 0.2% insulin). This 96-hour induction-maintenance cycle was repeated 3 times, after which cells were cultured in maintenance medium B for an additional 7 days. The remaining wells cultured in regular medium served as controls. Cells were refed with fresh differentiation medium in the differentiated wells and regular medium in control wells every 3 days. After 4 weeks of induction, Oil Red O staining (Sigma), which stained the lipid-laden vacuoles, was performed to assess the adipogenic differentiation potential of NPMSCs in each group. Briefly, wells were rinsed with PBS, fixed with 4% formaldehyde solution for 30 minutes, and then incubated in Oil Red O working solution for 30 minutes at room temperature (RT). After washing twice with PBS, wells were then visualized under fluorescence microscopy (ECLIPSE Ti-S, Nikon, Tokyo, Japan). Five fields of each well were randomly chosen and captured. Quantification was performed by determining the average percentage of total stained areas using ImageJ (NIH, Bethesda, MD, USA). This quantitative method was applied to further differentiation processes.

#### 4.7.2. Osteogenic Differentiation

P3 cells from the 2 age groups were harvested by trypsinization, seeded onto a 6-well plate at 3 × 10^4^ cells/cm^2^, and cultured in growth medium at 37°C in a 5% CO_2_ humidified incubator. After incubation for 24 hours, the medium of differentiated wells was gently aspirated and replaced with SD Rat Mesenchymal Stem Cell Osteogenic Differentiation Medium (Cyagen Biosciences, Guangzhou, China) containing 10% FBS, 1% penicillin-streptomycin, 0.01% dexamethasone, 1%  *β*-glycerophosphate, and 0.2% ascorbate. Cells cultured in regular medium served as controls and were refed every 3 days. After 3 weeks of induction, Alizarin Red staining (Sigma), which stained the calcified deposits, was performed to assess the osteogenic differentiation potential of young and old NPMSCs. Briefly, wells were rinsed with PBS, fixed with 4% formaldehyde solution for 30 minutes, and then stained with Alizarin Red solution for 5 minutes at RT. After washing several times with PBS, the stained areas in 5 fields of each well were visualized under fluorescence microscopy and then quantified using ImageJ as described above.

#### 4.7.3. Chondrogenic Differentiation

P3 cells from the 2 ages were harvested, seeded onto a 6-well plate at 2 × 10^4^ cells/cm^2^, and then maintained for 24 hours at 37°C in a 5% CO_2_ humidified incubator. After that, the growth medium of differentiated wells was replaced with SD Rat Mesenchymal Stem Cell Chondrogenic Differentiation Medium (Cyagen Biosciences, Guangzhou, China) containing 0.01% dexamethasone, 0.3% ascorbate, 1% ITS + supplement, 0.1% sodium pyruvate, 0.1% proline, and 1% TGF-*β*3. Cells cultured in standard medium served as controls and were refed every 3 days. After 3 weeks of induction, Alcian Blue staining (Sigma), which detected the synthesis of proteoglycans by chondrocytes, was performed to assess the chondrogenic differentiation potential of young and old NPMSCs. Briefly, wells were rinsed with PBS, fixed with 10% neutral buffered formalin, and then incubated in Alcian Blue solution for 30 minutes at RT. After washing several times with distilled water, the stained areas of a total of 5 fields per well were visualized under fluorescence microscopy and then quantified using ImageJ. For further assessing the potential of chondrogenesis, synthesis of other extracellular matrix components, such as collagen-II, aggrecan, and transcription factors, as Sox-9, was also detected by qPCR.

### 4.8. Cell Cycle Assay

The DNA content analysis was performed using the Cell Cycle and Apoptosis Analysis Kit (Beyotime Institute of Biotechnology, Haimen, China) according to the manufacturer's instructions. Briefly, 2 × 10^5^ cells from the 2 age groups were harvested at P3, washed twice with cold PBS, and then fixed in 70% prechilled ethanol for over 12 hours at 4°C. After that, cells were washed twice with cold PBS, incubated in propidium iodide (PI) staining buffer containing 200 mg/mL RNase A and 50 *μ*g/mL PI, and then stored for 30 minutes at 37°C in the dark. The intensity of fluorescence was determined at 488 nm by flow cytometry. Data were obtained by CellQuest software (BD, USA). The percentages of cells in the G0/G1, G2/M, and S phases were assessed by ModFit software (Verity Software House, Inc., Topsham, ME, USA).

### 4.9. Senescence-Associated *β*-Galactosidase (SA-*β*-gal) Staining

Both young and old NPMSCs were harvested at P3 and seeded onto a 12-well plate at 1 × 10^5^ cells/mL. We added 2 mL of cell suspension to each well and then incubated the cells at 37°C in a 5% CO_2_ humidified incubator. When cultured to 80%–90% confluence, cells were analyzed by the Senescence *β*-Galactosidase Staining Kit (Beyotime Institute of Biotechnology, China) following the manufacturer's protocol. Briefly, cells were washed with PBS, fixed in a SA-*β*-gal fixative solution for 15 minutes at RT, rinsed 3 times with PBS, and then incubated in the SA-*β*-gal working solution (reagents A, B, C, and X-Gal) overnight at 37°C without CO_2_. After that, 5 fields of each well were randomly selected and analyzed under fluorescence microscopy. Quantification was performed by determining the average percentage of total SA-*β*-gal-positive cells (300 cells per view).

### 4.10. Quantitative Real-Time Polymerase Chain Reaction (qPCR) Analysis

The expression of p53, p21, p16, collagen-II, aggrecan, and Sox-9 in young and old NPMSCs was determined by qPCR. Briefly, P3 cells and cells after induction of chondrogenesis were harvested, respectively. Total RNA was extracted using TRIzol reagent (Invitrogen Co., Carlsbad, CA, USA) according to the manufacturers' protocol. The purity of the total RNA was determined with an Ultraviolet Spectrometry Photometer. Then, the RNA was reverse-transcribed into cDNA with M-MLV Reverse Transcriptase (Promega, Madison, WI, USA). Briefly, 2 *μ*L of RNA was mixed with 2 *μ*L of 5x PrimeScript® RT MasterMix, and 10 *μ*L of RNase Free ddH_2_O was added. The mixed solutions were then incubated at 37°C for 15 minutes after 5 minutes at 70°C. Then, qPCR was performed following the instructions to determine the relative expressions of the target genes, and the results were normalized to the *β*-actin gene. The sequences of primers are listed in Table 1. The SYBR® Premix Ex Taq™ II (Tli RNaseH Plus) (Takara Bio, Otsu, Japan) was used for qPCR analysis. The qPCR consisted of an initial enzyme activation step at 95°C for 30 seconds, followed by 40 cycles of 95°C for 3 seconds and 60°C for 30 seconds. A cycle threshold (Ct) value was obtained for each sample, and triplicate sample values were averaged. The 2^−ΔΔCt^ value was then used to calculate the relative expression of each target gene. The data presented (means) were from 3 independent experiments in which both sample sets were analyzed in triplicate.

### 4.11. Statistical Analysis

Each experiment was performed at least in triplicate. Quantitative data are presented as the mean ± standard deviation (SD). Statistical analyses were performed using SPSS 15.0 software (Chicago, Illinois, USA). Student's *t*-test was used to assess the quantifiable data between groups. The Mann–Whitney *U* test was used to analyze the nonparametric data. A* P* value of <0.05 was considered statistically significant.

## Figures and Tables

**Table 1:** Primers utilized for real-time PCR amplification.

Target gene	Forward primer sequence	Reverse primer sequence
p53	5′-TACCGTATGAGCCACCTGAG-3′	5′-CAGGCACAAACACGAACCTC-3′
p21	5′-CAAAGTATGCCGTCGTCTGT-3′	5′-TCTCAGTGGCGAAGTCAAAG-3′
p16	5′-TACTCTCCTCCGCTGGGAAC-3′	5′-TGCCAGAAGTGAAGCCAAGG-3′
Collagen-II	5′-GGAAGAGTGGAGACTACTGGATTGAC-3′	5′-TCCATGTTGCAGAAAACCTTCA-3′
Aggrecan	5′-CCACTGGAGAGGACTGCGTAG-3′	5′-GGTCTGTGCAAGTGATTCGAG-3′
Sox-9	5′-AGGAAGCTGGCAGACCAGTACC-3′	5′-GGGTCTCTTCTCGCTCTCGTTCA-3′
*β*-Actin	5′-GGAGATTACTGCCCTGGCTCCTA-3′	5′-GACTCATCGTACTCCTGCTTGCTG-3′
